# Cantharidin and Its Analogue Norcantharidin Inhibit Metastasis—Inducing Genes S100A4 and MACC1

**DOI:** 10.3390/ijms24021179

**Published:** 2023-01-07

**Authors:** Paul Curtis Schöpe, Viktoria Zinnow, Muhammad Ahtisham Ishfaq, Janice Smith, Pia Herrmann, Robert H. Shoemaker, Wolfgang Walther, Ulrike Stein

**Affiliations:** 1 Experimental and Clinical Research Center, Charité—Universitätsmedizin and Max-Delbrück-Center for Molecular Medicine in the Helmholtz Association, 13125 Berlin, Germany; 2 Chemopreventive Agent Development Research Group, Division of Cancer Prevention, National Cancer Institute, Bethesda, MD 20892, USA; 3 German Cancer Consortium (DKTK Partnersite Berlin), Deutsches Krebsforschungszentrum (DKFZ), Im Neuenheimer Feld 280, 69120 Heidelberg, Germany

**Keywords:** colorectal cancer, metastasis, S100A4, MACC1, cantharidin, norcantharidin

## Abstract

Colorectal cancer (CRC) is the third most prevalent and second deadliest cancer worldwide. In addition, metastasis directly causes up to 90% of all CRC deaths, highlighting the metastatic burden of the disease. Biomarkers such as S100A4 and MACC1 aid in identifying patients with a high risk of metastasis formation. High expression of S100A4 or MACC1 and to a greater extent the combination of both biomarkers is a predictor for metastasis and poor patient survival in CRC. MACC1 is a tumor-initiating and metastasis-promoting oncogene, whereas S100A4 has not been shown to initiate tumor formation but can, nevertheless, promote malignant tumor growth and metastasis formation. Cantharidin is a natural drug extracted from various blister beetle species, and its demethylated analogue norcantharidin has been shown in several studies to have an anti-cancer and anti-metastatic effect in different cancer entities such as CRC, breast cancer, and lung cancer. The impact of the natural compound cantharidin and norcantharidin on S100A4 and MACC1 gene expression, cancer cell migration, motility, and colony formation in vitro was tested. Here, for the first time, we have demonstrated that cantharidin and norcantharidin are transcriptional inhibitors of S100A4 and MACC1 mRNA expression, protein expression, and motility in CRC cells. Our results clearly indicate that cantharidin and, to a lesser extent, its analogue norcantharidin are promising compounds for efficient anti-metastatic therapy targeting the metastasis-inducing genes S100A4 and MACC1 for personalized medicine for cancer patients.

## 1. Introduction

Colorectal cancer (CRC) was the third most common and second most deadly cancer worldwide in 2020 [1]. Most patients develop CRC after the age of 50 and up to 90% [2] of CRC deaths are attributed to the metastatic burden of the disease [3]. The low survival rate reflects the insufficient therapy options for metastasized patients. Major improvements for the survival rate of stage I–III patients have been made in the past decades, with little to no improvements for stage IV patients [4]. Therefore, effective biomarkers that identify patients with a high risk of metastasis, such as S100A4 and MACC1, as well as new therapies targeting the metastatic process are required.

S100A4 is a member of the calcium binding protein family with a size of 10–12 kDa and no known enzymatic function [5,6,7]. This small protein regulates or interacts with other proteins both intra- and extracellularly. Intracellular S100A4 interacts with proteins of the cytoskeleton such as actin, myosin, and tropomyosin and thereby increases directly cell motility [6,7] whereas extracellular S100A4 activates the expression of several matrix metalloproteinases. As a result, S100A4 enables cell invasion into adjacent tissues, facilitates the angiogenic process, and drives the metastatic process [3,4] due to its increased motility [4] and migratory properties [6]. Moreover, S100A4 is involved in other cellular functions such as differentiation and proliferation [5]. Although S100A4 can be associated with both malignant and non- malignant diseases [7], in cancer, S100A4 demonstrates enhanced cell growth and motility, tumor progression, and metastasis formation [7]. In addition, overexpression is associated with increased epithelial to mesenchymal transition (EMT) and chemoresistance [5].

High expression of S100A4 can be found in patient tumor tissues, blood, and circulating tumor cells in many different cancer entities such as CRC, lung, breast, and prostate cancer [5]. These properties make S100A4 a promising causative, prognostic, and predictive biomarker [8].

Mechanistically, S100A4 is the main target of the WNT/β-catenin signaling pathway which is significant since over 80% of CRC patients have mutated proteins which regulate this pathway. The signaling cascade can be disrupted by mutations in β-catenin itself, as well as by mutations in the destruction complex molecules or frizzled receptor leading to overexpression of S100A4 and increased invasiveness of the tumor. All in all, S100A4 is an excellent target for therapeutic interventions with multiple intervention possibilities at the transcriptional, translational, and post-translational level [7].

The gene metastasis-associated in colon cancer 1 (MACC1) was identified over a decade ago from a different display of RT-qPCR examining colon mucosa, primary tumors of stage I-III and stage IV, and metastases of subjects with colon cancer [9]. It was shown that the MACC1 expression level in tumor specimens is a stage-independent prognostic biomarker for metastasis formation and thus metastasis-free survival [10,11]. c-MET is one of MACC1 main transcriptional targets, causing proliferation, angiogenesis, and metastasis formation [9,11,12]. Since the discovery of MACC1, its unique structure, imbedding different protein interaction sites such as a SH3 domain, transcriptional activation, post-translational modifications, and its role in promoting nearly all cancer hallmark capabilities have been elucidated [10]. Furthermore, MACC1 has been recognized as a prognostic, predictive, and causative biomarker in more than 20 different cancer entities, including CRC, lung cancer, and gastric cancer making it a promising molecular target for solid cancers. Taken together, MACC1 represents a causal, prognostic, and predictive biomarker and promising molecular target for solid cancers [10,11].

The biotoxin cantharidin can be extracted from various species of the blister beetle and is considered as a cancer inhibitor in traditional Chinese medicine [13]. Moreover, cantharidin, although used for the treatment of warts and some parasites [14], is highly toxic in low concentrations [13]. The central oxygen atom within the benzene ring and the carboxylic acid anhydride residue was shown to be biochemically active. Cantharidin can inhibit proliferation [15], migration [14,16], invasion [16], and ultimately metastasis formation [17] in many different types of cancer such as breast, colon, and lung cancer [14]. In addition, it can induce apoptosis [14,15,16], cell cycle arrest [14,16], and autophagy [14]. The inhibitory effects on various cell signaling pathways such as MAPK, JNK, NFκ-B, and β-catenin [14] highlight cantharidin as a promising anticancer drug.

The demethylated analogue norcantharidin also displays strong bioactivity, with fewer side effects compared to cantharidin, however [16,18]. Norcantharidin likewise inhibits cell proliferation [19], migration [20], invasion [20], colony formation [21], and metastasis [22] in different cancer entities such as lung, breast, colon, and gastric cancer [20]. Similarly to cantharidin, norcantharidin induces apoptosis [1,3] and autophagy [1] and inhibits protein phosphatase 2A (PP2A) [20]. PP2A is an important serine threonine phosphatase which dephosphorylates several fundamental cellular molecules such as p53, c-Myc, and β-catenin, leading to the cell growth and survival of cancer cells [23]. Therefore, cantharidin and norcantharidin represent promising anti-tumor and anti-metastatic compounds.

A high-throughput screen (HTS) was conducted to search for S100A4 transcriptional inhibitors, employing a S100A4 promoter luciferase construct. Next to niclosamide, cantharidin was identified as a transcriptional inhibitor for S100A4 gene expression [24]. Thus, the aim of this study was to explore cantharidin and norcantharidin as transcriptional inhibitors for the metastasis inducing gene S100A4. In addition, since we recently elucidated the MACC1–β-catenin–S100A4 axis [25], we also will test the hypothesis of cantharidin/norcantharidin-inhibited MACC1 expression.

## 2. Results

### 2.1. Effect of Cantharidin and Norcantharidin on Cell Viability and Cytotoxicity

Cantharidin was found among the most effective hits in a HTS employing the S100A4 gene promoter linked to a reporter gene [24].

**Figure 1:**
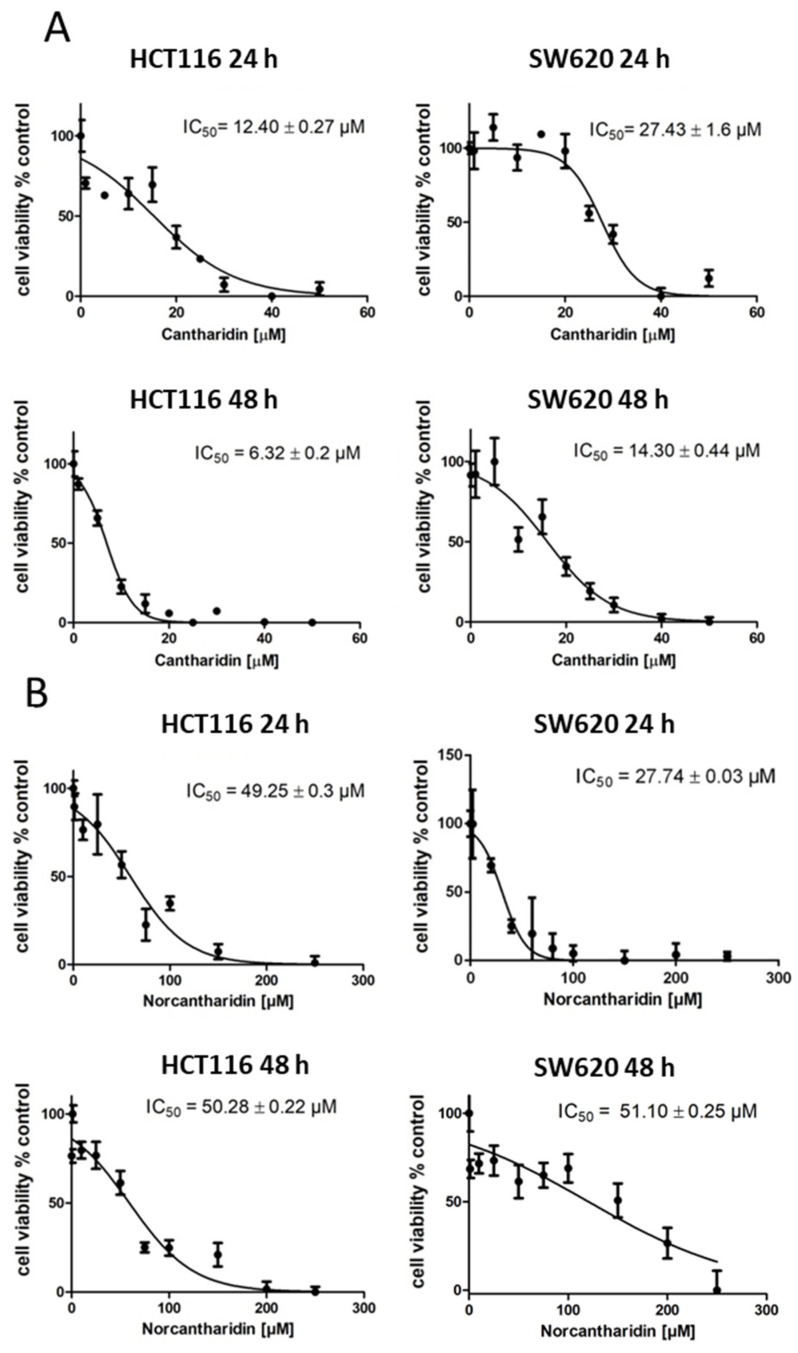
Assessment of cell viability measured using the MTT assay after 24 h and 48 h of treatment of increasing cantharidin or norcantharidin concentrations expressed as percent of DMSO control treated HCT116 or SW620 cells. The means and error bars represent three independent experiments. The IC_50_ values were calculated by nonlinear regression analysis. (**A**) Effect of cantharidin on cell viability in HCT116 and SW620 cells. **Top**: 24 h treatment and **bottom**: 48 h treatment. HCT116 cells on the left side and SW620 cells on the right side. (**B**) Effect of norcantharidin on cell viability in HCT116 and SW620 cells. **Top**: 24 h treatment and **bottom**: 48 h treatment. HCT116 cells on the left side and SW620 cells on the right side.

First, cell viability was tested in HCT116 and SW620 CRC cells using the MTT viability assay. Therefore, increasing concentrations of cantharidin and norcantharidin were applied to the cells for 24 h or 48 h (Figure 1). Cell viability decreased sharply with increasing cantharidin concentrations: HCT116 24 h: IC_50_ = 12.4 ± 0.27 µM; HCT116 48 h: IC_50_ = 6.32 ± 0.2 µM; SW620 24 h: IC_50_ = 27.43 ± 1.6 µM; SW620 48 h: IC_50_ = 14.30 ± 0.44 µM. In addition, cantharidin was confirmed to be more toxic in comparison to norcantharidin, reflected by a higher IC_50_ value of norcantharidin in both cell lines which stayed at similar levels after 24 h and 48 h treatment: HCT116 24 h: IC_50_ = 49.25 ± 0.3 µM; HCT116 48 h: IC_50_ = 50.28 ± 0.22 µM; SW620 24 h: IC_50_ = 27.74 ± 0.03 µM; SW620 48 h: IC_50_ = 51.10 ± 0.25 µM). In addition to the MTT assay, a LDH release assay was conducted to assess the cytotoxicity through membrane damage (Figures S1 and S2). The LDH assay confirmed the IC_50_ values determined with the MTT assay for cantharidin: HCT116 24 h: IC_50_ = 22.15 ± 1.05 µM; HCT116 48 h: IC_50_ = 6.323 ± 1.37 µM; SW620 24 h: IC_50_ = 13.90 ± 1.30 µM; SW620 48 h: IC_50_ = 8.131 ± 1.32 µM. For norcantharidin, little to no cytotoxicity was found using the same concentrations as in the MTT assay and the IC_50_ values could not be determined. Evaluation of the cytotoxicity data confirmed the higher toxicity of cantharidin compared to norcantharidin. Ten µM was identified as the optimal working concentration for both compounds and cell lines and was employed for subsequent functional assays.

### 2.2. Effect of Cantharidin and Norcantharidin on S100A4 and MACC1 mRNA and Protein Expression

**Figure 2:**
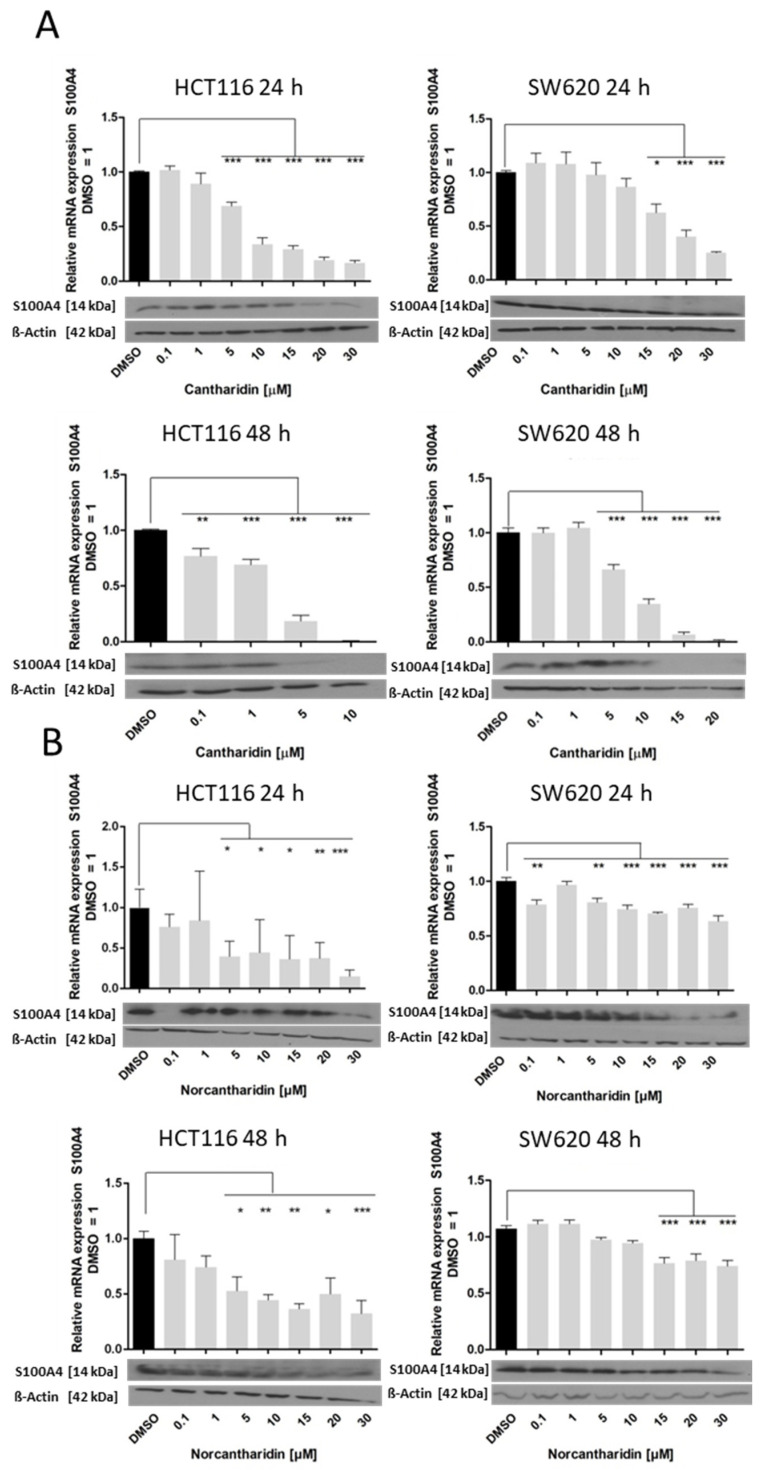
Effect of cantharidin and norcantharidin on S100A4 mRNA and protein expression after 24 h and 48 h. The HCT116 and SW620 cells were treated daily with increasing concentrations (0.1–30 µM) vs. the DMSO control. RNA was extracted and RT-qPCR was conducted for the S100A4 mRNA represented as the percentage of the DMSO control of three independent experiments. The black bar indicates the DMSO control and the grey bars are the experimental samples which represent the data mean ± SEM (*n* = 3), * *p* < 0.05, ** *p* < 0.01, *** *p* < 0.001. Protein levels were analyzed with an immunoblot assay with β-actin as a loading control. The blots represent one of the three independent experiments. (**A**) Effect of cantharidin on S100A4 mRNA and protein expression in HCT116 and SW620 cells. **Top**: 24 h treatment and **bottom**: 48 h treatment. HCT116 cells left side, SW620 cells right side. (**B**) Effect of norcantharidin on S100A4 mRNA and protein expression in HCT116 and SW620 cells. **Top**: 24 h treatment and **bottom**: 48 h treatment. HCT116 cells on the left side and SW620 cells on the right side.

**Figure 3:**
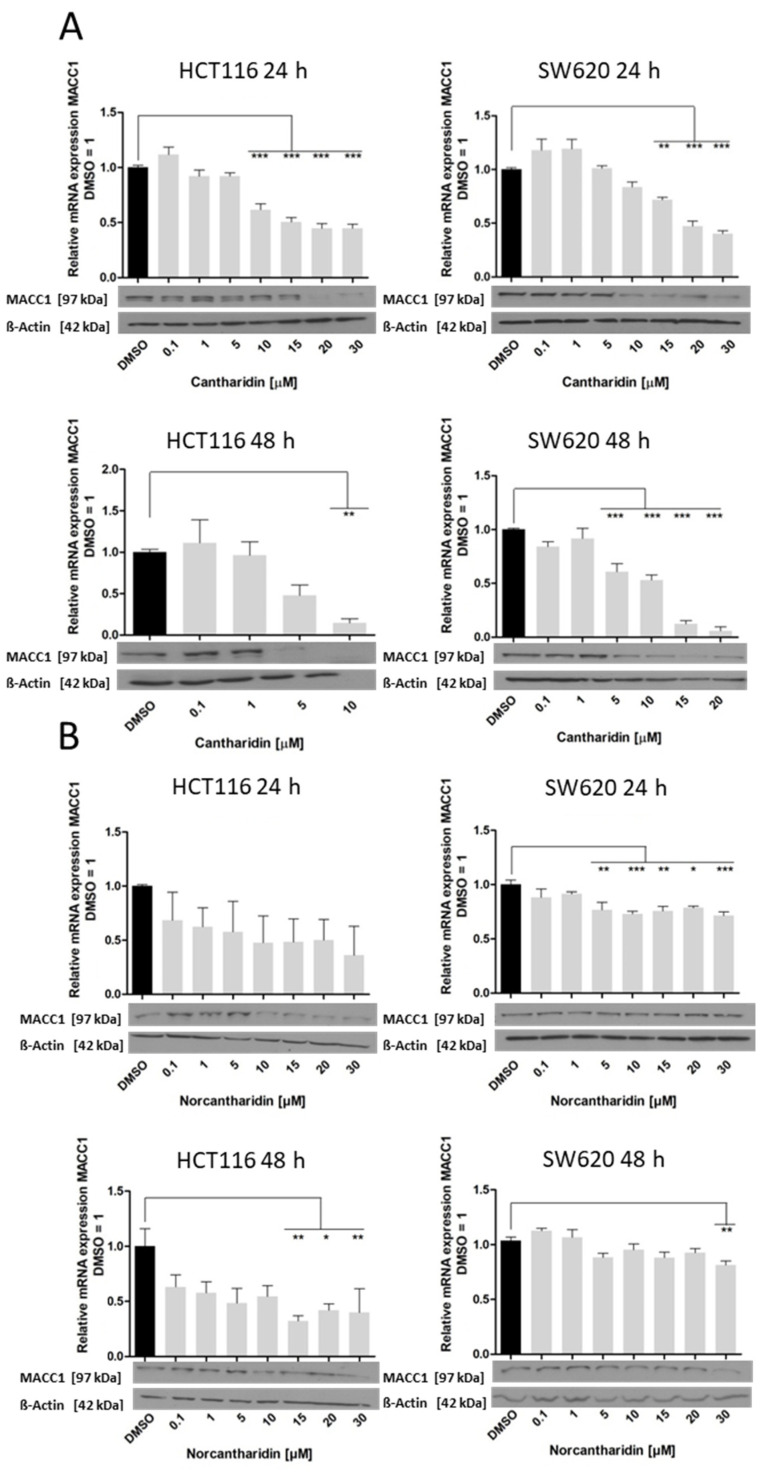
Effect of cantharidin and norcantharidin on MACC1 mRNA and protein expression after 24 h and 48 h. HCT116 and SW620 cells were treated daily with increasing concentrations (0.1–30 µM) vs. the DMSO control. RNA was extracted and RT-qPCR was conducted for the MACC1 mRNA represented as a percentage of the DMSO control of three independent experiments. The black bar indicates the DMSO control and the grey bars are the experimental samples which represent data mean ± SEM (*n* = 3), * *p* < 0.05, ** *p* < 0.01, *** *p* < 0.001. Protein levels were analyzed by immunoblot assay with β-actin as a loading control. The blots represent one of the three independent experiments. (**A**) Effect of cantharidin on MACC1 mRNA and protein expression in HCT116 and SW620 cells. **Top**: 24 h treatment and **bottom**: 48 h treatment. HCT116 cells on the left side and SW620 cells on the right side. (**B**) Effect of norcantharidin on MACC1 mRNA and protein expression in HCT116 and SW620 cells. **Top**: 24 h treatment and **bottom**: 48 h treatment. HCT116 cells on the left side and SW620 cells on the right side.

Next, the effect of cantharidin and norcantharidin on the S100A4 (Figure 2) and MACC1 (Figure 3) mRNA and protein expressions were analyzed with increasing concentrations (0.1–30 µM) in both cell lines. HCT116 cells and SW620 cells showed decreasing S100A4 mRNA and protein levels with increasing cantharidin or norcantharidin concentrations after 24 h and 48 h (Figure 2). Treatment of HCT116 cells with 10 µM of cantharidin or norcantharidin resulted in a more than 50% inhibition of S100A4 mRNA expression. For the SW620 cells, a strong effect was seen at 10 and 15 µM concentrations of both compounds. This inhibitory effect is greater in HCT116 cells compared to SW620 cells. Cantharidin exhibited stronger cytotoxicity after 48 h of treatment, resulting in a smaller concentration panel for HCT116 and SW620 cells; HCT116: 0.1–10 µM; SW620: 0.1–20 µM. The strongest effect was seen for HCT116 cells treated with cantharidin and the lowest effect was observed in SW620 cells treated with norcantharidin.

Next, the effect of cantharidin and norcantharidin on the gene expression of MACC1 was evaluated (Figure 3). Interestingly, a similar effect as that observed for the S100A4 gene was observed. MACC1 gene expression decreased in a concentration-dependent manner after 24 h and 48 h of treatment with cantharidin or norcantharidin in both cell lines. Cantharidin reduced MACC1 mRNA expression in the HCT116 cells by 50% after treatment with 15 µM and 5 µM after 24 h and 48 h, respectively. For the SW620 cells, inhibition of more than 50% was observed at 20 µM and 15 µM after 24 h and 48 h, respectively. MACC1 protein expression was inhibited after treatment with 20 µM and 5 µM cantharidin for 24 h and 48 h, respectively, for both cell lines. Norcantharidin showed a similar trend compared to the S100A4 gene. In HCT116 cells, norcantharidin reduced MACC1 gene expression by 50% at 10 µM for both time points, whereas MACC1 protein expression was inhibited at the highest concentration of 30 µM. For the SW620 cell line, a less prominent effect was seen with strong inhibition above 50% at 10 µM after 24 h and 30 µM after 48 h. The inhibitory effect on gene expression was then translated into decreased protein expression, showing a band of lower intensity after treatment with a 30 µM concentration.

### 2.3. Time-Dependent Effect of Cantharidin and Norcantharidin on S100A4 and MACC1 Gene Expression Levels

**Figure 4:**
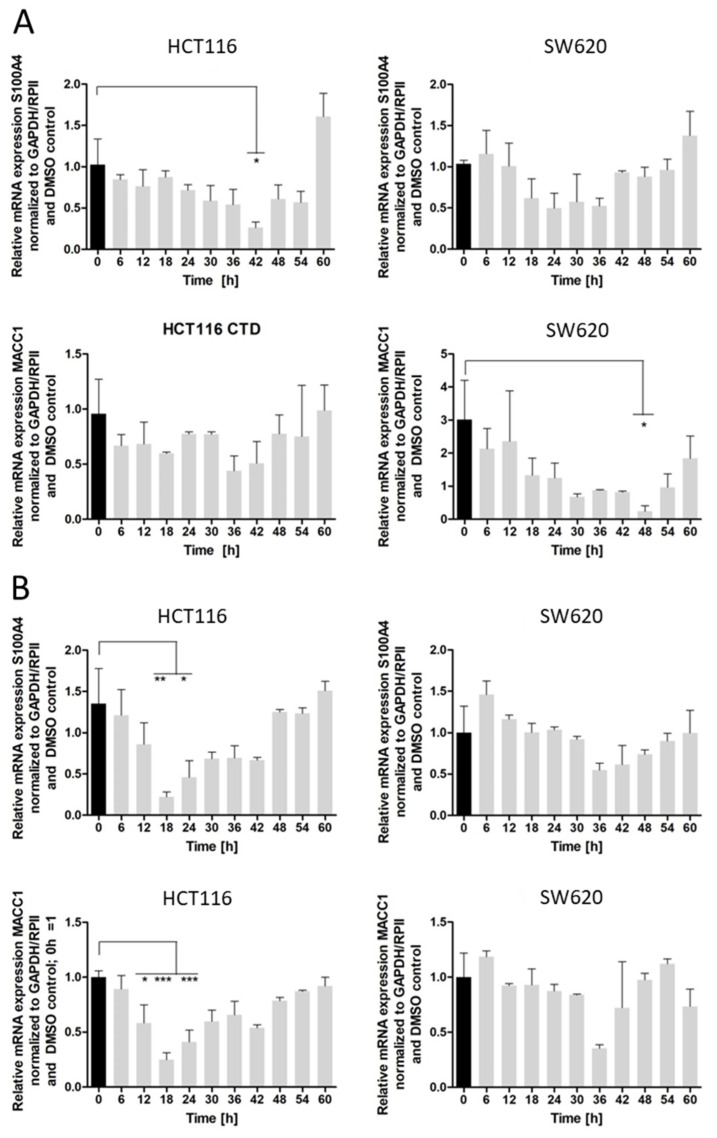
Time-dependent analysis of gene expression inhibition by cantharidin (**A**) and norcantharidin (**B**) of S100A4 (**top**) and MACC1 (**bottom**) in HCT116 (**left**) and SW620 (**right**) cells. The cells were treated once with 10 µM of cantharidin or norcantharidin and every 6 h the samples were taken over a time course of 60 h. RNA was extracted and RT-qPCR was conducted for S100A4, MACC1, and the control genes GAPDH and RPII. The black bar indicates the starting point at 0 h and the grey bars indicate each timepoint after 0h. The data represent mean ± SEM (*n* = 3), * *p* < 0.05, ** *p* < 0.01, *** *p* < 0.001. (**A**) Time dependent inhibition of S100A4 gene (**top**) and MACC1 gene (**bottom**) expression after treatment of HCT116 (**left**) and SW620 cells (**right**) with 10 µM of cantharidin. (**B**) Time-dependent inhibition of S100A4 gene (**top**) and MACC1 gene (**bottom**) after treatment of HCT116 (**left**) and SW620 cells (**right**) with 10 µM of norcantharidin.

Next, we evaluated the cantharidin- and norcantharidin-induced, time-dependent inhibition of S100A4 and MACC1 gene expression (Figure 4). The cells were treated once with a single concentration of 10 µM compound and then the mRNA expression levels were analyzed every 6 h for 60 h. Cantharidin effects are shown for HCT116 (left panel) and SW620 (right panel) cells, with S100A4 in the top panel and MACC1 in the lower panel (Figure 4A). Cantharidin inhibited the gene expression of S100A4 for 42 h in HCT116 cells and for 36 h in SW620 cells. Furthermore, a similar trend was seen for the MACC1 gene expression with 36 h of inhibition in HCT116 cells and with 48 h of inhibition in SW620 cells.

The time-dependent effect of norcantharidin on S100A4 is shown in the top panel and the time-dependent effect of the MACC1 gene is shown in the bottom panel in HCT116 (left panel) and SW620 cells (right panel) (Figure 4B). Norcantharidin also reduced S100A4 and MACC1 mRNA expression in HCT116 cells with an inhibition peak at 18 h for both genes. In SW620 cells, weaker mRNA reduction was seen, with an inhibition peak at the 36 h time point.

The gene expression inhibition, however, ends for both singular applications of cantharidin and norcantharidin in HCT116 and SW620 cells, with the latest inhibition at 54 or 60 h following the start of treatment.

### 2.4. Effect of Cantharidin and Norcantharidin on Colony Formation and Migration

**Figure 5:**
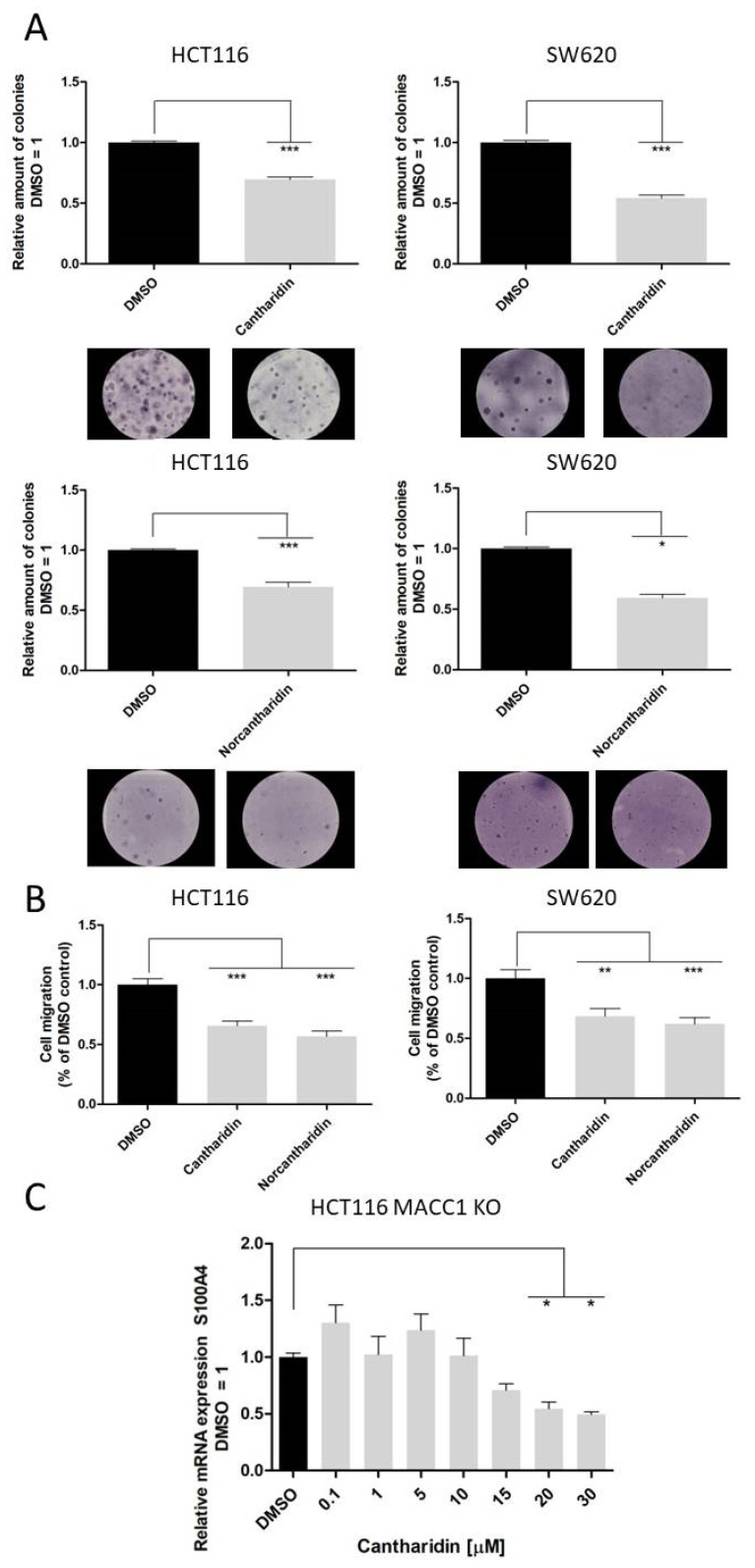
Effect of cantharidin and norcantharidin on anchorage-independent colony formation and cell migration. (**A**) Anchorage-independent colony formation. The cells were plated as single cells in 0.33% (wt/vol) agarose and treatment was performed with solvent or 10 µM cantharidin or a norcantharidin-containing medium. After 7 days, colonies were visualized by light microscopy and counted for colony formation (>4 cells = 1 colony). Magnification = 10×. (**B**) Cell migration of cantharidin- or norcantharidin-treated cells (treatment as in (**A**)). Cell migration was determined using Boyden chamber assay and expressed as percent of solvent-treated cells. (**C**) Effect of cantharidin on gene expression of S100A4 in HCT116 KO MACC1 cells. The means and 95% confidence intervals from the three independent experiments are presented in all of the panels. The data represent mean ± SEM (*n* = 3), * *p* < 0.05, ** *p* < 0.01, *** *p* < 0.001.

We next analyzed the functions that are mediated by the S100A4 and MACC1 genes, namely colony formation and the migration of cells. The evaluation of anchorage-independent growth by colony formation was carried out by seeding single cells in 0.33% (wt/vol) agarose and treating them with 10 µM of either cantharidin or norcantharidin vs. the DMSO control. After 7 days, colonies were visualized and counted using a light microscope. Colony formation following treatment with cantharidin (top) and norcantharidin (bottom) is shown for HCT116 (left) and SW620 (right) cells (Figure 5A). Both compounds inhibited colony formation compared to the DMSO control. Cantharidin inhibited the colony formation by 30% in HCT116 cells and 46% in SW620 cells HCT116: DMSO vs. cantharidin, mean = 100% vs. 70%, mean difference = 0.303 ± 0.024, 95% CI = 0.254 to 0.354. *p* < 0.001; SW620: DMSO vs. cantharidin, mean = 100% vs. 54%, mean difference = 0.459 ± 0.0316, 95% CI = 0.388 to 0.529, *p* < 0.001. Furthermore, the size of the colonies was reduced under cantharidin treatment. A similar effect was observed after norcantharidin treatment with 31% inhibition in HCT116 and 41% in SW620 cells HCT116: DMSO vs. norcantharidin, mean = 100% vs. 69%, mean difference = 0.306 ± 0.040, 95% CI = 0.221 to 0.392. *p* < 0.001; SW620: DMSO vs. norcantharidin, mean = 100% vs. 59%, mean difference = 0.409 ± 0.033, 95% CI = 0.339 to 0.478, *p* < 0.001. Taken together, cantharidin and norcantharidin inhibited the colony formation ability of both cell lines in comparison to the vehicle control (DMSO).

Furthermore, S100A4 and MACC1 are important regulators of cell motility [3,4,10]. Hence, a Boyden chamber assay was conducted to analyze the cell migratory ability under cantharidin and norcantharidin treatment, using a single concentration of 10 µM and a vehicle control (DMSO) (Figure 5B). Both compounds inhibited migration compared to the DMSO control. Cantharidin inhibited migration by 34% in HCT116 cells and 32% in SW620 cells; HCT116: DMSO vs. cantharidin, mean = 100% vs. 66%, mean difference = 0.3431, 95% CI = 0.1995 to 0.4867. *p* < 0.001; SW620: DMSO vs. cantharidin, mean = 100% vs. 68%, mean difference = 0.3161, 95% CI = 0.1115 to 0.5207. *p* < 0.001. Norcantharidin inhibited migration by 43% in HCT116 and 38% in SW620 cells; HCT116: DMSO vs. norcantharidin, mean = 100% vs. 57%, mean difference = 0.4320, 95% CI = 0.2884 to 0.5756. *p* < 0.001; SW620: DMSO vs. norcantharidin, mean = 100% vs. 62%, mean difference = 0.380, 95% CI = 0.1754 to 0.5846. *p* < 0.001.

As shown previously, S100A4 is transcriptionally activated by MACC1 [25]. To test the possibility of cantharidin being a sole MACC1 inhibitor, inhibiting S100A4 via MACC1, we used HCT116 KO MACC1 cells (Figure 5C). Treating HCT116 KO MACC1 cells with cantharidin resulted in a reduction in S100A4 at the mRNA level, with inhibition of 50% at the 20 µM concentration. This shows that the reduction in S100A4 gene expression by cantharidin is independent of MACC1 signaling.

## 3. Discussion

The aim of this study was to evaluate cantharidin- and norcantharidin- induced inhibition of gene expression in the metastasis genes S100A4 and MACC1 on a transcriptional level. Two colorectal cancer cell lines with different intrinsic S100A4 and MACC1 gene expression levels were used: HCT116 cells with moderate expression levels of S100A4 and MACC1 and SW620 with high expression levels of S100A4 and MACC1 [25].

In this study, we identified cantharidin as a more effective drug in terms of mRNA and protein expression inhibition of S100A4 and MACC1 in comparison to norcantharidin. In addition, mRNA expression inhibition by both compounds was significantly higher in HCT116 cells compared to SW620 cells. As SW620 cells were extracted from a lymph node metastasis, they are likely to hold a higher metastatic potential compared to the HCT116 cell line. The gene expression levels of S100A4 and MACC1 are also generally higher in SW620 cells, potentially needing a higher concentration of the compound to inhibit gene expression in a similar manner compared to HCT116 cells.

Nevertheless, both drugs inhibit S100A4 and MACC1 expression, with significantly higher inhibition of S100A4 expression. We recently showed that MACC1 is able to regulate the gene expression of S100A4 [25], therefore to rule out a MACC1-mediated reduction in S100A4 mRNA levels, we also used the HCT116 KO MACC1 cell line. In the MACC1 knock-out set-up, the effect of cantharidin on the S100A4 gene remains comparable, pointing to a dual effect of cantharidin and norcantharidin on both S100A4 and MACC1 expression (Figure 5). Concerning the functional consequences of S100A4 and MACC1 inhibition, colony formation and migration ability were greatly impaired by both drugs. Even a modest reduction of mRNA and protein levels of S100A4 and MACC1 is able to reduce the number of migrating cells in our assay.

The inhibition of colony formation and migration, however, was comparable between cantharidin and norcantharidin. Nonetheless, norcantharidin is substantially less toxic than cantharidin. The norcantharidin concentration applied for the time-dependent and function-related assays was chosen based on cantharidin remaining consistent and comparable between both drugs; however, a higher concentration of norcantharidin could be applied due to its lower toxicity level to reach similar effects. Lower compound concentrations are usually more beneficial to avoid toxicity. Furthermore, cantharidin shows a longer-lasting inhibition of mRNA expression of both genes after a single treatment compared with norcantharidin.

Compared to known inhibitors of S100A4, such as niclosamide [24], a higher concentration of cantharidin and norcantharidin is needed to achieve a similar inhibitory effect on the gene expression; however, this effect is longer lasting for cantharidin as niclosamide inhibition peaks at 18–24 h. Therefore, treatment every 48 h is possible for cantharidin for in vivo or clinical trials, leading to less stress for animals and patients caused by treatment applications.

Furthermore, cantharidin inhibits both metastasis genes to a greater extent than norcantharidin, exemplified by a greater inhibition on both the mRNA and protein level and a longer lasting effect in the time-dependent experiments. Since these two drugs differ only by two missing methyl groups, it can be assumed that the demethylation of norcantharidin plays a significant role in its effect on gene expression and cell viability. Both drugs interfere with known gene pathways such as the WNT and NFκ-B signaling pathways [14,16]. Cantharidin is known to inhibit protein phosphatase 2A (PP2A) [15] which in turn leads to a more active destruction complex in the WNT/β-catenin signaling pathway. This then leads to lower activation of this specific pathway by degrading β-catenin, a transcriptional activator of WNT target genes [7]. In addition to MACC1-activated transcription, the WNT signaling pathway also plays a major role in the transcriptional activation of the S100A4 gene [7,25]. Furthermore, the NFκ-B signaling pathway can lead to higher transcriptional activity of the MACC1 gene [26]. Inhibition of PP2A can inhibit the activation of the NFκ-B pathway by reduced activation of the IKKα complex, which could lead to reduced MACC1 gene expression. Moreover, the MAPK pathway with ERK as an effector molecule is the most analyzed pathway leading to higher MACC1 gene expression with c-MET on top of the signaling cascade. AP-1 and SP-1 transcription factors can bind to and regulate the MACC1 promoter as downstream molecules of the MAPK pathway [10]. The MAPK pathway has been shown to be modulated by cantharidin in breast cancer cells leading to reduced cell growth and migration [16]. All of the mentioned pathways play an important role in metastasis formation by increasing the proliferation and motility of cancer cells and are likely to be involved in the mode of action of cantharidin and norcantharidin which needs further investigation and experimental validation in further studies, as it is beyond the scope of this study.

In summary, while cantharidin is a more cytotoxic compound compared to norcantharidin, it has a greater inhibitory effect on the metastasis genes S100A4 and MACC1. Here, for the first time, we have shown that cantharidin and to a lesser extent norcantharidin inhibits the mRNA expression of S100A4 and MACC1 and the cellular functions mediated by both metastasis genes such as colony formation and migration. This study contributes to the understanding of the inhibitory effect of both compounds on the development of invasive cells and metastasis formation. Based on S100A4 and MACC1 expression, patients at high risk of developing metastasis can be identified. In this context, both compounds might be a valuable therapeutic option for these patients in a personalized medicine setting. This can be achieved as monotherapy with these drugs or as combination therapy with other metastasis inhibitors such as niclosamide or with cytostatic drugs, such as 5-FU. Moreover, Xu et al. [27] showed that through combinatorial treatment with angiogenic inhibitors, the anti-cancer effect of cantharidin was released. Careful consideration needs to be taken and further investigations for synergistic effects need to be carried out for combinatorial treatment with cantharidin for in vivo and human clinical trials.

## 4. Materials and Methods

### 4.1. Cell Lines and Cell Culture

The human CRC cell lines HCT116 and HCT116 KO MACC1 were grown in RPMI 1640 medium supplemented with 10% (vol/vol) fetal bovine serum and the human CRC cell line SW620 was grown in DMEM medium supplemented with 10% (vol/vol) fetal bovine serum without any antibiotics. All of the cell lines were expanded in T75 flasks, passaged twice a week and incubated at 37 °C with 5% CO_2_. The HCT116 and SW620 cell lines were initially purchased from the American Type Culture Collection (ATCC, Manassas, VA, USA). The HCT116 KO MACC1 cells were created by B. Kortüm as described previously for SW620 cells [25]. Furthermore, all of the cell lines were tested routinely every two weeks for mycoplasma contamination using the Mycoalert mycoplasma detection kit (Lonza, Basel, Switzerland).

### 4.2. High throughput Screening (HTS)

HTS was performed as previously described [24]. Briefly, the HCT116 cells used expressed firefly luciferase under CMV-promoter control (HCT116/CMVpLUC cells) or under control of the S100A4-promoter (comprising the sequence from −1487 bp upstream- to the S100A4 transcription start site; HCT116/S100A4pLUC cells). The S100A4 promoter sequence was a kind gift from David Allard (Peninsula Medical School, University of Exeter and University of Plymouth, Exeter, UK). The HCT116 cells were transfected with the S100A4 cDNA, kindly provided by Claus Heizmann (University of Zurich, Zurich, Switzerland; HCT116/S100A4 cells) or the empty vector as the control (HCT116/vector cells). Stable transgene expressing cells were selected with 1 mg/mL neomycin (PAA Laboratories, Cölbe, Germany) or 1 µg/mL puromycin (Invitrogen, Carlsbad, CA, USA).

2.5 × 10^3^ cells/well of HCT116/S100A4pLUC cells were seeded in 384-well plates, and the cells were treated for 24 h with each compound of the LOPAC 1280 library. Luciferase expression was determined by Britelite reagent (Perkin Elmer, Waltham, MA, USA). In parallel, the cytotoxicity of the compounds was measured by the AlamarBlue*™* cytotoxicity assay (AbD Serotec, Raleigh, NC, USA). Reporter inhibition efficacy was determined by the ratio of toxicity versus activity.

### 4.3. Monoclonal and Polyclonal Antibodies

The polyclonal rabbit anti-human S100A4 antibody was purchased from Dako (Glostrup, Denmark) and the polyclonal anti-human MACC1 antibody was purchased from Sigma-Aldrich (Schnelldorf, Germany). The mouse anti-human β-actin antibody was purchased from Sigma-Aldrich (Germany). The goat anti-mouse IgG antibody was purchased from R&D Systems (Mineapolis, MN, USA) and the mouse anti-rabbit IgG HRP conjugate was purchased from Promega (Madison, WI, USA).

### 4.4. Drugs and Treatments

Cantharidin and norcantharidin were obtained from Sigma-Aldrich. All of the drugs were dissolved in DMSO which was used as a solvent control. After 24 h incubation of the cells, the drug treatment was started by removing the old medium (only for 6- and 24-well plates) and adding a fresh medium containing the respective compounds, and all of the drug dilutions contained the same amount of solvent; the 48 h treatments were treated twice every 24 h. Only for the 96-well plates was no medium removed prior to the drug treatment. Afterwards, the plates were incubated for the respective time in a humidified incubator at 37 °C and 5% CO_2_.

### 4.5. MTT Assay

Cell viability was measured via an MTT assay. One hundred μL with 1 × 10^4^ cells were seeded on a 96-well plate and incubated overnight at 37 °C. The next day, the drug treatment was prepared in double concentrations and 100 μL of each drug concentration were added to each well and incubated for 24 h or 48 h. Afterwards, 20 μL of MTT (final concentration: 0.5 mg/mL) solution was added to the wells and incubated for 1 h, followed by removal of 120 μL of medium, addition of 100 μL of SDS lysis buffer (10% SDS, 1 mM HCl), and incubation overnight. Cell viability was measured using a plate reader and the internal software (Fluor Spectra Plus, Tecan, Männedorf, Switzerland) at 560 nm the following day.

### 4.6. LDH Cytotoxicity Assay

Cytotoxicity was measured via an LDH release assay using the CyQuant LDH Cytotoxicity Assay (Invitrogen). Briefly, 1 × 10^4^ cells were seeded on a 96-well plate and incubated overnight at 37 °C. The next day, the drug treatment was prepared in double concentrations and 100 µL of each drug concentration was added to each well and incubated for 24 h or 48 h. The control wells were set up according to the manufacturer’s instructions. The next day the LDH levels were measured in the supernatant in accordance with the manufacturer’s instructions. The wavelengths at 490 and 680 nm were measured with a plate reader (Infinite Series 2000). The cytotoxicity was calculated as instructed by the manufacturer.

### 4.7. Expression Experiments

#### 4.7.1. RNA Extraction

1 × 10^5^ cells were seeded in 24-well plates. After 24 h of incubation, the cells were treated. After incubation, the medium was removed and RNA extraction using the universal RNA extraction kit from Roboklon (Berlin, Germany) was conducted according to the manufacturer’s instructions. RNA was stored at −80 °C.

#### 4.7.2. Quantitative Reverse Transcription–Polymerase Chain Reaction (qRT-PCR)

The samples from the RNA purification were measured with a Nanodrop using its internal software (nanodrop 1000, Preqlab, Darmstadt, Germany), mRNA integrity was measured by dividing the wavelength of 260/280 nm, and 50 ng of RNA was used in the reverse transcription. A master mix consisting of 5.25 μL PCR-grade water, 2 μL 5× buffer, 1 μL dNTPs, 0.25 μL (40 U/µL) RNase inhibitors, 0.5 μL (200 U/µL) reverse transcriptase, and 0.5 μL (25 µM) of hexamer primers was prepared. The reverse transcription was administrated as following using a Mastercycler (Eppendorf (Hamburg, Germany), Mastercylcer Nexus Gradient): 10 min at 30 °C, 40 min at 50 °C, and 5 min at 99 °C. The reverse transcription tubes were stored at −20 °C. The S100A4 and MACC1 mRNA expression analyses were carried out with the LightCycler system 2.0 and its internal software (Roche Diagnostics, Basel, Switzerland). As a calibrator, the DNA of SW620 colorectal cancer cells were used to perform a standard curve which served to correlate the target gene concentration for each run. GAPDH and RP II served as housekeeper control genes. The qPCR master mix consists of 0.4 µL of the forward and reverse primer, 5 µL Cyber Green Mastermix, and 2.2 µL PCR-grade water per well. The PCR was administrated as following: 30 s at 95 °C, 40 times 5 s at 95 °C, and 20 s at 60 °C and a melting curve with a continuous temperature increase from 65 to 95 °C with a rate of 0.1 °C/s. Each sample was run in triplicates. The LightCycler 480 software release 1.5.0 SP3 (Roche Diagnostics) was used for analysis.

#### 4.7.3. Protein Extraction and Immunoblot

5 × 10^5^ cells were seeded in six-well plates and after 24 h of incubation, drug treatment was conducted. To stop the experiment, the cells were pelleted and stored at −80 °C. For lysing the cells, RIPA buffer supplemented with protease inhibitors was added to the cell pellet on ice for 30 min with vortexing every 10 min. After preparation, the samples were centrifuged at 4 °C for 30 min at 14.800× *g*. The protein concentrations were measured using the BCA assay kit (Pierce BCA Assay kit, Thermo Scientific, Waltham, MA, USA) according to producers’ instructions. Forty µg were separated using sodium dodecyl sulfate-polyacrylamide gel electrophoresis (SDS-PAGE) with 12.5% gel with 70 V for 30 min and 120 V until the end. After the separation, the proteins were transferred onto an activated PVDF membrane by semi-dry blotting (Trans-blot turbo transfer system, Bio-Rad, CA, USA). To block the membranes, they were incubated with 5% non-fat dry milk for 1 h at room temperature with light shaking. Afterwards, the membranes were washed once with TBS-T and were cut according to the size of the proteins. Next, all of the membranes were incubated overnight at 4 °C with a rabbit anti-human S100A4 antibody (dilution, 1:400), a rabbit anti-human MACC1 antibody (dilution, 1:3000), and mouse anti-human beta-Actin (dilution, 1:40,000) as a reference. The next day, the membranes were washed 6 × 5 min with TBS-T followed by incubation with a HRP-conjugated anti-rabbit antibody (dilution, 1:10,000) for S100A4 and the MACC1 protein and a goat anti-mouse antibody (dilution 1:40,000) for beta-Actin for 1 h at room temperature. At the end, the membranes were washed 6 × 5 min with TBS-T and the protein–antibody complexes were visualized by Western Bright Peroxide and Western Bright ECL (dilution, 1:1) and exposed to x-ray films.

#### 4.7.4. Boyden Chamber Transwell Migration

The migration ability of HCT116 cells was analyzed by a Boyden chamber transwell migration assay. For this, at least 4 × 10^6^ cells were seeded in a Petri dish with 10% FBS medium and incubated overnight. The next morning, the cells were washed with PBS and a medium without FBS was added and incubated for 5 h. After that, serum-free cells were counted and 1 × 10^6^ cells/mL in medium with 0.5% FBS and the respective drug concentrations were seeded in the upper part of the Boyden chamber well plate. In the bottom chamber, the drug and medium with 10% FBS were added. After 16 h, the migrated cells in the bottom chamber were collected with 0.05% Trypsin/EDTA and counted using a Neubauer counting chamber. The treatments were normalized to the DMSO control.

#### 4.7.5. Colony Formation assay

The soft agar colony formation assay was used for the analysis of anchorage-independent cell proliferation. For the bottom layer, 2 mL of 0.5% (wt/vol) agarose, RPMI-1640 medium, 10% FBS, and 10 µM cantharidin, norcantharidin, or the respective volume of DMSO were added to a 3 cm culture dish and incubated at room temperature under sterile conditions for 10 min. On top of the solidified bottom layer, HCT116 and SW620 cells (8 × 10^3^ cells) were added in 0.33% (wt/vol) agarose, RPMI-1640-medium, 10% FBS, and 10 µM cantharidin, norcantharidin, or the respective volume of DMSO. After that, the culture dishes were incubated for 7 days at 37 °C and 5% CO_2_ in a humidified incubator. Visualization was performed using 10× magnification for an overview and 40× for the single colonies in the Leica DMIL light microscope (Leica Microsystems, Wetzlar, Germany). Colonies with more than four cells were counted in 10 squares of 1 µm^2^. The experiments were repeated three times independently, each in triplicate.

### 4.8. Statistical Analysis

All of the statistics were performed with GraphPad Prism version 5.0 (La Jolla, CA, USA). Gaussian distribution was tested using the Kolmogorov–Smirnov test. For analyzing the three groups, one-way analysis of variance (ANOVA) with Dunne’s post-hoc test was performed. For comparison of the two groups, a two-tailed *t*-test was performed. *p* values smaller than 0.05 were considered as statistically significant (* = *p* < 0.05, ** = *p*< 0.01, *** = *p* < 0.001).

## 5. Conclusions

In conclusion, our study presents for the first time cantharidin and norcantharidin as inhibitors of the metastasis-inducing genes S100A4 and MACC1. Both compounds inhibit the mRNA and protein expression of S100A4 and MACC1 as well as S100A4- and MACC1-mediated functions, such as colony formation and migration. Cantharidin showed a longer lasting inhibitory effect on S100A4 and MACC1 mRNA compared to norcantharidin. Ultimately, cantharidin has higher toxicity with a higher efficacy of gene expression inhibition of S100A4 and MACC1.

## Data Availability

The data presented in this study are available on request from the corresponding author.

## Supplementary Materials

The following supporting information can be downloaded at: https://www.mdpi.com/article/10.3390/ijms24021179/s1.
