# Supplementary material for: Cantharidin and Its Analogue Norcantharidin Inhibit Metastasis—Inducing Genes S100A4 and MACC1
Source: Int J Mol Sci. 2023 Jan 7;24(2):1179. doi: 10.3390/ijms24021179 (PMC9866560; doi:10.3390/ijms24021179)
Supplement: Supplementary file 1 [file ijms-24-01179-s001.zip › ijms-2053064-supplementary.pdf]

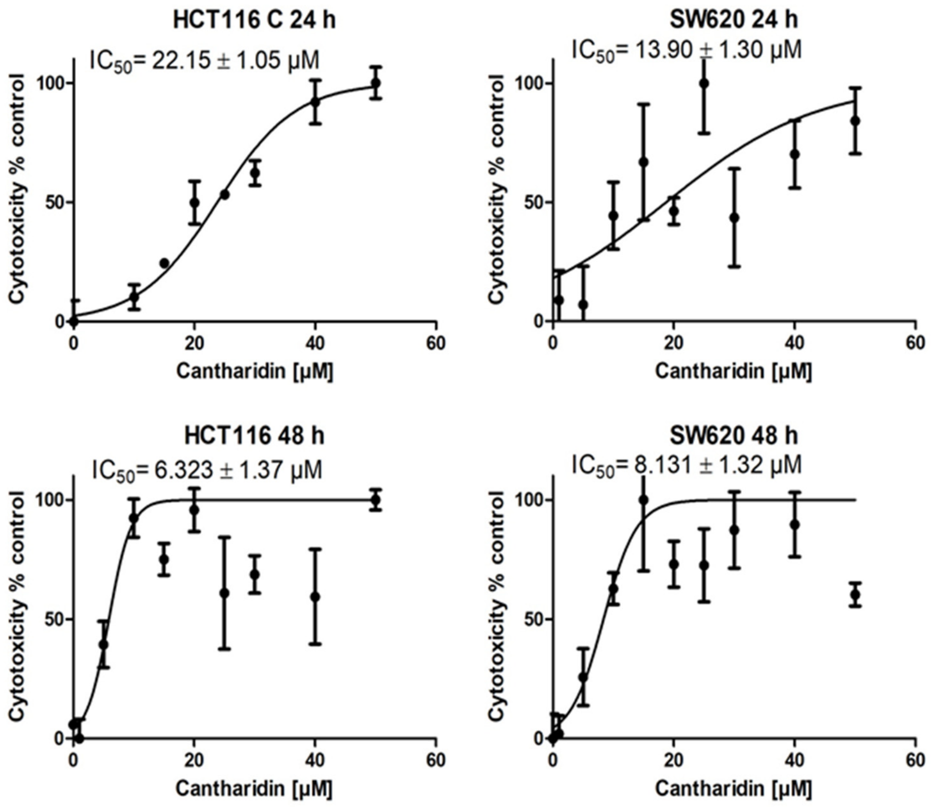

Figure S1: Assessment of cytotoxicity measured using LDH release assay after 24 h (top) and 48 h (bottom) treatment of increasing cantharidin concentrations expressed as percent of cytotoxicity of control treated HCT116 (left) or SW620 (right) cells. The means and error bars represent 3 independent experiments. IC_{50} values were calculated by nonlinear regression analysis.

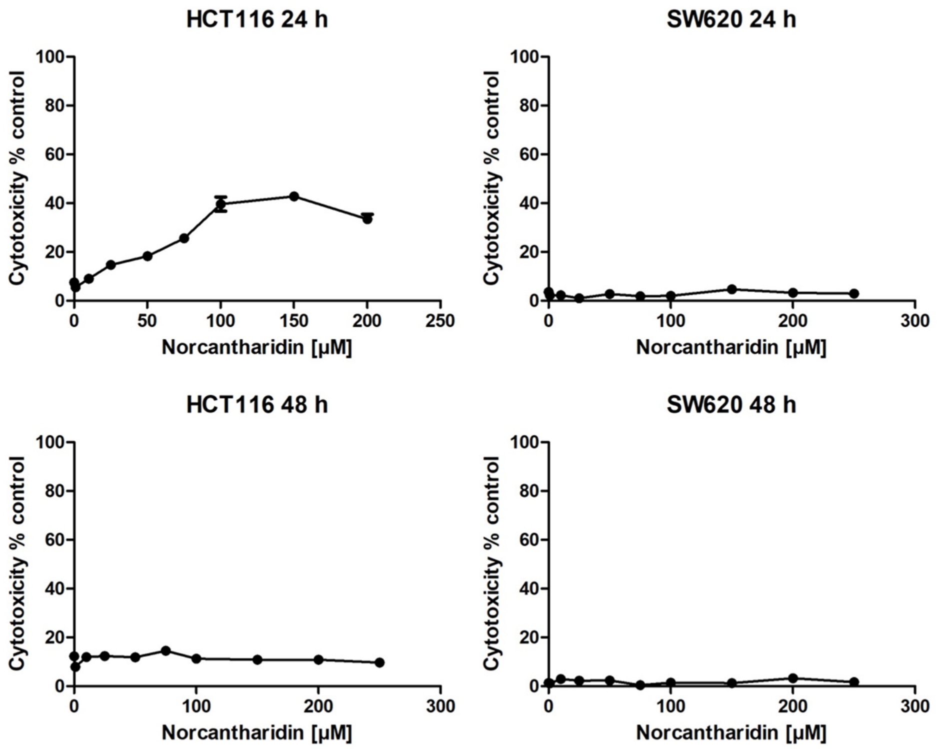

Figure S2: Assessment of cytotoxicity measured using LDH release assay after 24 h (top) and 48 h (bottom) treatment of increasing norcantharidin concentrations expressed as percent of cytotoxicity of control treated HCT116 (left) or SW620 (right) cells. The means and error bars represent 3 independent experiments.
